# Experimental investigation of oil–water flow in the horizontal and vertical sections of a continuous transportation pipe

**DOI:** 10.1038/s41598-021-99660-8

**Published:** 2021-10-11

**Authors:** Jianlei Yang, Peng Li, Xuhui Zhang, Xiaobing Lu, Qing Li, Lifei Mi

**Affiliations:** 1 Hebei Petroleum University of Technology, Chengde, 067000 China; 2 Institute of Mechanics, Chinese Academy of Sciences, Beijing, 100190 China; 3 School of Engineering Science, University of Chinese Academy of Sciences, Beijing, 100049 China; 4 Engineering and Construction Department, Dagang Oilfield, Tianjin, 300280 China; 5 Oil Production Technology Institute, Dagang Oilfield, Tianjin, 300280 China

**Keywords:** Petrol, Fluid dynamics

## Abstract

A series of experiments were conducted to investigate flow pattern transitions and concentration distribution during simultaneous pipe flow of oil–water two-phase flow through the horizontal and vertical sections. The flowing media applied were white mineral oil and distilled water. Superficial oil and water velocities were between 0 and 0.57 m/s. Flow pattern maps revealed that the horizontal and vertical sections of the pipe lead to different flow pattern characteristics under the same flow conditions. The original contributions of this work are that a transition mechanism for predicting the boundary between oil-in-water (O/W) flow and water-in-oil (W/O) in oil–water two-phase flow was obtained. The effects of input water cut, oil and water superficial velocities on the concentration distribution of the dispersed phase were studied. The empirical formulas for the phase holdup based on the drift-flux model were obtained. The predicted results agreed well with those of the experimental data, especially for the O/W flow pattern.

## Introduction

With the increase of oil well production life, the volume fraction of water in oil pipes may gradually increase^1^. The oil–water two-phase flow in the pipe is a crucial topic in the petroleum industry. The oil–water flow is characterized by a variety of diverse flow patterns. Different concentration and pressure drop distribution characteristics occurred under different flow patterns. The understanding of flow pattern and phase holdup is of great importance for the evaluation of oilfield operating and oil production. The change of the flow pattern has a vital impact on the pressure drop, spatial phase distribution, and safety of pipeline transportation^2^. The calculation of water holdup is helpful to predict the oil transportation in oil pipelines, and water holdup is an important parameter in identifying flow patterns^3^.

Many researchers have been studied the simultaneous oil and water flow in horizontal or vertical pipes. For horizontal oil–water flows, the flow patterns are mainly divided into the segregated flow and the dispersed flow. The segregated flow did not occur in pipes with an inclination of more than 33^·^. The oil–water flow often occurs as the dispersed flow^4^. Piela et al.^5^ studied the phase inversion of oil–water flow experimentally through a horizontal pipe loop. The experiments were carried in a pipe loop with an inner pipe diameter of 16 mm, consisting of two straight parts of 6 m connected via two bends. Experimental data showed that the phase inversion occurred at the oil volume fraction of 0.9 for the water-to-oil inversion, and the pressure drop increases at the inversion compared to the initial pressure drop. The volume fraction of the dispersed phase at the water-to-oil experiment transition point was higher than that for oil-to-water experiments. Gong et al.^6^ developed a model for predicting phase inversion in oil–water flow in a stainless steel pipe loop using the relations between the surface energies before and after phase inversion. The results of the model were in reasonable agreement with the experimental results. The experiments were conducted in a stainless steel pipe loop (25.7 mm inner diameter, 52 m long). The experimental temperature was 40 °C, 50 °C, and 60 °C, respectively. Kumara et al. measured an oil–water two-phase flow in a horizontal pipe with a diameter of 56 mm and length of 15 m with particle image velocimetry and laser Doppler anemometry^7^. Zhou et al. designed parallel-wire conductivity probes to measure water hold-up of oil–water two-phase flow in a near-horizontal pipe. The experimental data showed that the method can achieve a good measurement result of water hold-up^8^. Wang et al. conducted oil–water experiments in a small horizontal channel with 2 mm. Flow patterns and the plug shape/length taken by highspeed camera were investigated^9^.

For vertical oil–water systems, Hasan and Kablr^10^ adopted the drift-flux model to predict in situ oil volume fraction in oil–water flow. The model parameters were determined by the experimental data. The experimental rig consisted of two separate 5.5 m long transparent columns with 64 and 127 mm inner diameters. Xu et al.^11^ conducted an experimental study of a co-current upward and downward in a transparent vertical pipe. The phase inversion and frictional pressure gradients were investigated using white oil and water in a 50 mm inner diameter pipe. The flow patterns are usually recognized by visual inspection and the fluctuations in the volume fraction. Keska et al.^12^ carried out the comparative experimental research on four methods (capacitive, resistive, optical, and pressure) commonly adopted to identify the flow pattern, and the results proved that the best way to judge the flow pattern is to use the capacitance or resistance fluctuation of the flow cross-section. The values of resistance and capacitance represent the change in the volume fraction. Jones and Zuber^13^ demonstrated that the probability density function (PDF) of the fluctuations in the volume fraction can be used as a statistical analysis tool for flow pattern identification. The PDF is a function of signal amplitude and a method of measuring the probability that a signal has a range of values. In this study, the PDF of the volume fraction of the water phase is used to identify changes in the flow pattern. Du et al. employed convolutional neural networks to identify oil–water two-phase flow patterns. The different flow pattern images were collected through oil-in-water flow experiments in a vertical 20 mm inner diameter Plexiglas pipe. The flow pattern images were collected by the highspeed camera^14^.

However, few comparative studies of horizontal and vertical flows in a combined pipe have been reported. In the on-site oil pipeline layout, the pipeline is horizontal, vertical, or even inclined. Under the same flow parameters, pipelines in different directions may show different flow pattern characteristics, which brings hidden dangers to the stability of pipeline transportation. Under the same flow parameters, it is necessary to study the different flow behaviors in horizontal and vertical pipelines^3^.

The oil–water flow in the pipe is complex and related to pipe geometries (e.g., inner pipe diameter, pipe angle), fluid properties (e.g., viscosity, density, and surface tension), and boundary conditions (e.g., superficial input velocities). Previous studies mainly focused on the effect of a single parameter, but the coupled effect of the controlling parameters on the flow is not well understood. Hence, to understand the fundamental mechanisms of oil–water flows, the controlling dimensionless parameters were derived by dimensional analysis first.

Thus, the objective of this work was to investigate the flow pattern transition and phase holdup for an oil–water two-phase flow through the horizontal and vertical sections, a comparative study of the flow behavior has been conducted in a pipe loop by using different superficial input velocities. A series of experiments were conducted to investigate the oil–water flow pattern transition and the water holdup, considering both the horizontal and vertical sections of a transportation pipe.

The current paper is structured as follows. The experimental setup is described in “Experimental setup and procedure” section. The dimensional analysis is presented in “Dimensional analysis and data structure” section. The overall results in terms of flow pattern and phase holdup are discussed in “Results and discussion” section. The relationship between the transient water holdup and the change of the flow pattern in a transportation pipe with horizontal and vertical sections is established, and the empirical formulas for the phase holdup based on the drift-flux model are obtained. Finally, “Conclusion” section presents the conclusions of this study.

## Experimental setup and procedure

### Experimental facility

**Figure 1:**
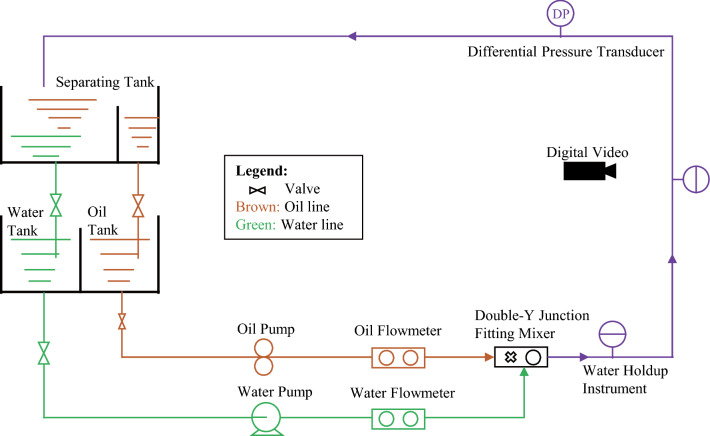
Schematic drawing of the flow loop used in this study.

The experimental study for oil–water flow was conducted in a closed circuit loop. The schematic drawing of the flow loop is depicted in Fig. 1, which can be used to carry out both oil–water or oil–water–gas flow experiments. The test equipment can identify the oil–water flow patterns and record the water holdup (in situ volume fraction of water) and the pressure drop.

The flowing pipe was composed of a stainless steel pipe section and a plexiglass pipe section, and its inner diameter was 50 mm. The flow pattern development section was a horizontal stainless steel pipe with a length of 10 m from the entrance (corresponding to 200*D*, where *D* was the inner diameter). Based on the experimental observation, this length ensured that a fully developed flow can be achieved before measurement^6^. Due to the mixture velocities were low (*u* < 0.57 m/s) and oil phase viscosity high (32 mPa s), this length provided sufficient length to stabilize the flow. Flores^2^ also used the development length of about 200 diameters to study the characterization of oil–water flow patterns. The reasonableness of the development length is further confirmed in “Flow pattern maps” section.

The flow observation sections consisted of a 1 m long horizontal transparent pipe and a 2 m long vertical transparent pipe. With this arrangement, experiments on horizontal and vertical flows can be carried out simultaneously. The water was pumped first by a centrifugal pump (QABP160M2A, ABB), with a capacity of 12.5 m^3^/h. Then the oil phase was pumped into the clapboard mixer in which the oil was mixed with the water. A 6.99 KW gear pump (SNH440) with a capacity of 17 m^3^/h and accuracy of ± 0.1% of the reading was used for the oil phase. The oil and water phases entered the double-Y junction fitting mixer from the upper and lower layers of the mixer, respectively. The angle of the meeting point of the oil and water flow lines before entering the mixer is 60 degrees. The oil–water mixture flowed simultaneously to the test section and then flowed down the return section to the separating tank. Leaving the separating tank, the oil and water were directed to their original tanks. The liquid volume flow rate was monitored by a mass flowmeter (CMF100, Micro Motion), whose accuracy was ± 0.1%.

### Measuring equipment

Measurements included water holdup, pressure drop, and flow patterns, in addition to recording parameters such as the volume flow rate of each phase and the experimental temperatures. A differential pressure transducer was installed in the return section to measure the pressure drop at a frequency of 1 Hz. When the pressure drop was independent of time, it was deemed that a steady state of the system was reached. Then a total of 300 s of data was recorded. A digital video was used to observe the flow structures and identify the flow patterns. The working fluids used in the experiments were white mineral oil and distilled water. All experiments were carried out under atmospheric pressure, and the room temperature was controlled at about 20 °C. The physical parameters of the oil phase and water phase are shown in Table 1.

**Table 1:** Properties of water and oil phases measured at atmospheric pressure and 20 °C.

	Density, $\rho$ (kg/m^3^)	Viscosity, $\mu \;({\text{mPa}}\;{\text{s}})$	Interfacial tensions, $\sigma \;({\text{mN}}\;{\text{m}})$
White mineral oil	843	32	–
Distilled water	998.2	1	Water/oil, 42

**Figure 2:**
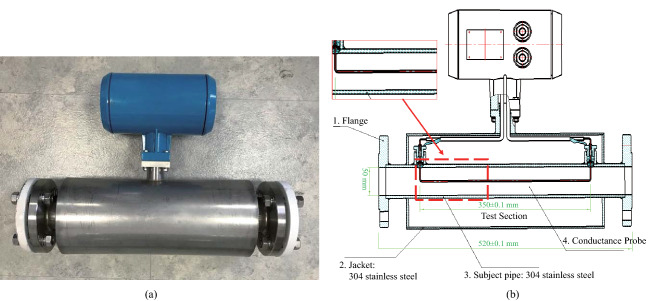
(**a**) Photograph of the water holdup instrument designed for the experiments. (**b**) The internal structure of the water holdup instrument^3^.

Two water holdup instruments equipped with a conductance probe were assembled in the horizontal and vertical sections of the test pipe to measure the cross-sectional average water holdup $\alpha_{{\text{w}}}$ at a sampling frequency of 1 Hz, respectively. The photograph of the water holdup instrument is shown in Fig. 2^3^. The conductance probe measured the voltage between the two ends of the conductor, which allowed the mean conductivity of the mixture in the pipe to be calculated. Due to the poor conductivity of the oil phase, the voltage value measured when the pipe was filled with pure oil was much higher than that when the pipe was filled with pure water. The voltage values of pure oil and water were denoted by *V*_o_ and *V*_w,_ respectively. Then, the mean conductivity *V*_exp_ of the in-suit liquid can be determined by the following equation:

1 $$ V_{\exp } = f\left( {\alpha_{{\text{w}}} ,\;V_{{\text{w}}} ,\;V_{{\text{o}}} } \right) $$

Taking *V*_w_ as a unit system produced the following dimensionless relationship:

2 $$ \frac{{V_{\exp } }}{{V_{{\text{w}}} }} = f\left( {\alpha_{{\text{w}}} ,\frac{{V_{{\text{o}}} }}{{V_{{\text{w}}} }}} \right) $$

According to the mixture theory^15^, it was assumed that the voltage value of the mixture satisfied the following relationship:

3 $$ \frac{{V_{\exp } }}{{V_{{\text{w}}} }} = \alpha_{{\text{w}}} + \left( {1 - \alpha_{{\text{w}}} } \right)\frac{{V_{{\text{o}}} }}{{V_{{\text{w}}} }} $$

The relationship between water holdup $\alpha_{{\text{w}}}$ and the mean conductivity of the mixture *V*_exp_ can be expressed as:

4 $$ \alpha_{{\text{w}}} = \frac{{V_{{\text{o}}} - V_{\exp } }}{{V_{{\text{o}}} - V_{{\text{w}}} }} $$

Noting that the voltage values of pure water and pure oil were slightly different under different experimental conditions. Therefore, both *V*_o_ and *V*_w_ adopt the values measured under the current experimental condition when the voltage value signal was converted into the water holdup using Eq. (4).

## Dimensional analysis and data structure

The flow patterns of oil–water two-phase flow are classically organized into the separated flow and dispersed flow^4^. The dispersed flow frequently occurs in oil–water flow, where one phase is dispersed phase, and the other is continuous phase. These two types of dispersion spontaneously invert at some operational conditions, which is called phase inversion. A single uppercase subscript *C* refers to the continuous phase and *D* refers to the dispersed phase. The parameters controlling the oil–water two-phase dispersed flow are listed as follows:

Dispersed phase: density $\rho_{D}$, viscosity $\mu_{D}$, drop diameter $d_{D}$, interfacial tension $\sigma$.

Continuous phase: density $\rho_{C}$, viscosity $\mu_{C}$.

Geometric parameter: inner pipe diameter *D*.

Boundary condition: superficial input velocity $u_{s}$ which is defined as $u_{s} = Q{/}A$ where *Q* is the volume flow rate of each phase and *A* is the cross-sectional area of the pipe, $A = \pi D^{2} /4$.

Gravitational acceleration: *g*.

The eventual steady state of the oil–water flow system, characterized by the holdup of the dispersed phase $\alpha_{D}$, pressure drop $\Delta P$, and flow pattern, is a function of the above controlling parameters:

5 $$ \left( {\alpha_{D} ,\;\Delta P,\;flow\;pattern} \right) = f\left( {\rho_{C} , \, \mu_{C} , \, u_{sC} ; \, \rho_{D} , \, \mu_{D} , \, u_{sD} , \, d_{D} , \, \sigma ; \, D, \, g} \right) $$

Equation (5) can be nondimensionalized as:

6 $$ \left( {\alpha_{D} ,\;\frac{\Delta P}{{\rho_{C} u_{sC}^{2} }}, \, flow\;pattern} \right) = f\left( {\frac{{u_{sD} }}{{u_{sC} }}, \, \frac{{\rho_{C} u_{sC} D}}{{\mu_{C} }}, \, \frac{{u_{sC}^{2} }}{gD}, \, \frac{{\left| {\rho_{C} - \rho_{D} } \right|gD^{2} }}{\sigma }, \, \frac{{\mu_{D} }}{{\mu_{C} }}, \, \frac{{\rho_{D} }}{{\rho_{C} }}, \, \frac{{d_{D} }}{D}} \right) $$

where $u_{sD} /u_{sC}$ is the superficial velocity ratio, $\rho_{C} u_{sC} D/\mu_{C}$ is Reynolds number $Re_{C}$, $u_{sC}^{2} /gD$ is Froude number *FrC*. ${{\left( {\left| {\rho_{C} - \rho_{D} } \right|gD^{2} } \right)} \mathord{\left/ {\vphantom {{\left( {\left| {\rho_{C} - \rho_{D} } \right|gD^{2} } \right)} \sigma }} \right. \kern-\nulldelimiterspace} \sigma }$ is Eötvös number *Eo* that represents the ratio of buoyancy force to surface tension force. For a wholly developed dispersed flow, the velocity difference between oil and water phases is minimal. Then, in the present paper, the dimensionless numbers are calculated instead of the superficial velocity $u_{sC}$, with the total superficial velocity $u_{{s{\text{m}}}}$.

7 $$ u_{{s{\text{m}}}} = u_{sC} + u_{sD} $$

Defining input water cut $\varepsilon_{{\text{w}}}$ as the ratio of the water flow rate to the mixture flow rate.

8 $$ \varepsilon_{{\text{w}}} = \frac{{Q_{{\text{w}}} }}{{Q_{{\text{w}}} + Q_{{\text{o}}} }} = \frac{{u_{{s{\text{w}}}} }}{{u_{{s{\text{m}}}} }} $$

Assuring that the fluid properties and drop size remain constant, i.e., Eötvös number *Eo*, density ratio $\rho_{D} /\rho_{C}$, viscosity ratio $\mu_{D} /\mu_{C}$ , and the ratio of drop size to pipe diameter $d_{D} /D$ are fixed. Therefore, the Eq. (6) can be modified as:

9 $$ \left( {\alpha_{D} ,\;\frac{\Delta P}{{\rho_{C} u_{{s{\text{m}}}}^{2} }},\; \, flow\;pattern} \right) = f\left( {\varepsilon_{{\text{w}}} , \, \frac{{\rho_{C} u_{{s{\text{m}}}} D}}{{\mu_{C} }}, \, \frac{{u_{{s{\text{m}}}}^{2} }}{gD}} \right) $$

The effects of the dimensionless parameters in Eq. (9) on the flow characteristics of the oil–water two-phase flow are investigated. All the experimental values of $u_{{s{\text{w}}}}$, $u_{{s{\text{o}}}}$, and the corresponding dimensionless numbers are given in Table 2. Noting that $Re_{{\text{m}}}$ is not listed in this table and calculated according to which phase is continuous in the experiments. The material-related dimensionless parameters remain constant.

**Table 2:** Data structure for the oil–water pipe flow experiments ($Eo = 90.53$, $\mu_{{\text{o}}} /\mu_{{\text{w}}} = 32$, $\rho_{{\text{o}}} /\rho_{{\text{w}}} = 0.84$).

Test	$Q_{{\text{w}}}$ (m^3^/s)	$Q_{{\text{o}}}$ (m^3^/s)	$u_{{s{\text{w}}}}$ (m/s)	$u_{{s{\text{o}}}}$ (m/s)	$\varepsilon_{{\text{w}}}$	$u_{{s{\text{m}}}}^{2} /gD$
1	0	44.44 × 10^−5^	0.00	0.23	0	0.108
2	4.44 × 10^−5^	40.00 × 10^−5^	0.02	0.20	0.1	0.108
3	8.89 × 10^−5^	35.56 × 10^−5^	0.05	0.18	0.2	0.108
4	13.33 × 10^−5^	31.11 × 10^−5^	0.07	0.16	0.3	0.108
5	17.78 × 10^−5^	26.67 × 10^−5^	0.09	0.14	0.4	0.108
6	22.22 × 10^−5^	22.22 × 10^−5^	0.11	0.11	0.5	0.108
7	26.67 × 10^−5^	17.78 × 10^−5^	0.14	0.09	0.6	0.108
8	31.11 × 10^−5^	13.33 × 10^−5^	0.16	0.07	0.7	0.108
9	35.56 × 10^−5^	8.89 × 10^−5^	0.18	0.05	0.8	0.108
10	40.00 × 10^−5^	4.44 × 10^−5^	0.20	0.02	0.9	0.108
11	44.44 × 10^−5^	0	0.23	0.00	1.0	0.108
12	0	63.89 × 10^−5^	0.00	0.33	0	0.222
13	6.39 × 10^−5^	57.50 × 10^−5^	0.03	0.29	0.1	0.222
14	12.78 × 10^−5^	51.11 × 10^−5^	0.07	0.26	0.2	0.222
15	19.17 × 10^−5^	44.72 × 10^−5^	0.10	0.23	0.3	0.222
16	25.56 × 10^−5^	38.33 × 10^−5^	0.13	0.20	0.4	0.222
17	31.94 × 10^−5^	31.94 × 10^−5^	0.16	0.16	0.5	0.222
18	38.33 × 10^−5^	25.56 × 10^−5^	0.20	0.13	0.6	0.222
19	44.72 × 10^−5^	19.17 × 10^−5^	0.23	0.10	0.7	0.222
20	51.11 × 10^−5^	12.78 × 10^−5^	0.26	0.07	0.8	0.222
21	57.50 × 10^−5^	6.39 × 10^−5^	0.29	0.03	0.9	0.222
22	63.89 × 10^−5^	0	0.33	0.00	1.0	0.222
23	0	111.11 × 10^−5^	0.00	0.57	0	0.663
24	11.11 × 10^−5^	100.00 × 10^−5^	0.06	0.51	0.1	0.663
25	22.22 × 10^−5^	88.89 × 10^−5^	0.11	0.45	0.2	0.663
26	33.33 × 10^−5^	77.78 × 10^−5^	0.17	0.40	0.3	0.663
27	44.44 × 10^−5^	66.67 × 10^−5^	0.23	0.34	0.4	0.663
28	55.56 × 10^−5^	55.56 × 10^−5^	0.28	0.28	0.5	0.663
29	66.67 × 10^−5^	44.44 × 10^−5^	0.34	0.23	0.6	0.663
30	77.78 × 10^−5^	33.33 × 10^−5^	0.40	0.17	0.7	0.663
31	88.89 × 10^−5^	22.22 × 10^−5^	0.45	0.11	0.8	0.663
32	100.00 × 10^−5^	11.11 × 10^−5^	0.51	0.06	0.9	0.663
33	111.11 × 10^−5^	0	0.57	0.00	1.0	0.663

## Results and discussion

### Flow pattern maps

The experiments of wash-out type oil–water two-phase pipe flow were carried out by starting with pumping water first and then adding oil applying different flow rates. The identification of flow regimes was based on both visual observation and interpretation of transient flow signals (e.g., fluctuations of water holdup and pressure drop). Flores^4^ mainly identified water-dominated pattern (dispersion of oil in water, O/W) and oil-dominated pattern (dispersion of water in oil, W/O) for oil–water flow in vertical and horizontal pipes. The present work focused on the phase inversion between oil-in-water and water-in-oil dispersions.

**Figure 3:**
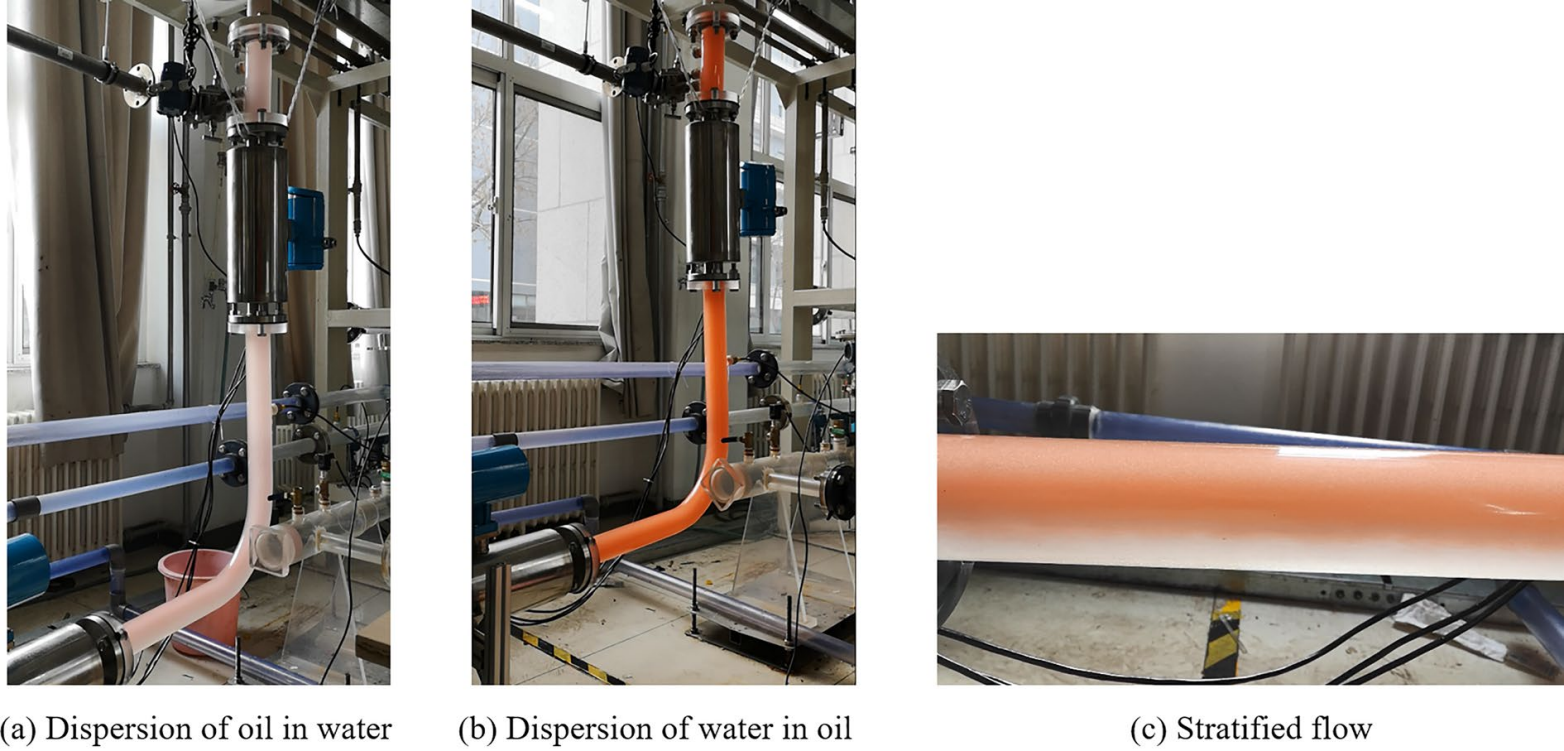
Instantaneous flow patterns observed in the experiments in the horizontal and vertical sections: (**a**) dispersion of oil in water, $u_{{s{\text{w}}}} = 0.16$ m/s, $u_{{s{\text{o}}}} = 0.07$ m/s; (**b**) dispersion of water in oil, $u_{{s{\text{w}}}} = 0.02$ m/s, $u_{{s{\text{o}}}} = 0.20$ m/s; (**c**) stratified flow, $u_{{s{\text{w}}}} = 0.40$ m/s, $u_{{s{\text{o}}}} = 0.17$ m/s.

Figure 3 shows examples of the flow patterns that were observed in this study in both the horizontal and vertical sections in a continuous transportation pipe. Figure 3a presents the O/W pattern. Figure 3b shows the W/O pattern, where a liquid film with a certain thickness formed between the gas column and the pipe wall. Figure 3c shows the stratified flow. The upper layer was the continuous oil phase and the lower layer was the continuous water phase. Based on the experimental observations, the development length can achieve the fully developed flow patterns.

**Figure 4:**
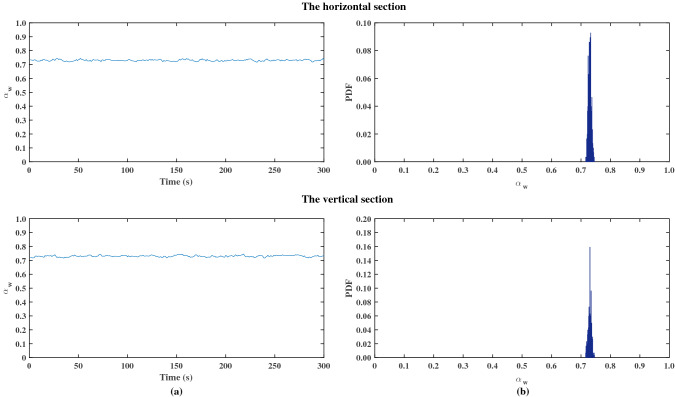
Typical evolution of the cross-sectional average water holdup $\alpha_{{\text{w}}}$ and PDF for oil–water pipe flow: dispersion of oil in water ($u_{{s{\text{w}}}} = 0.16$ m/s, $u_{{s{\text{o}}}} = 0.07$ m/s).

**Figure 5:**
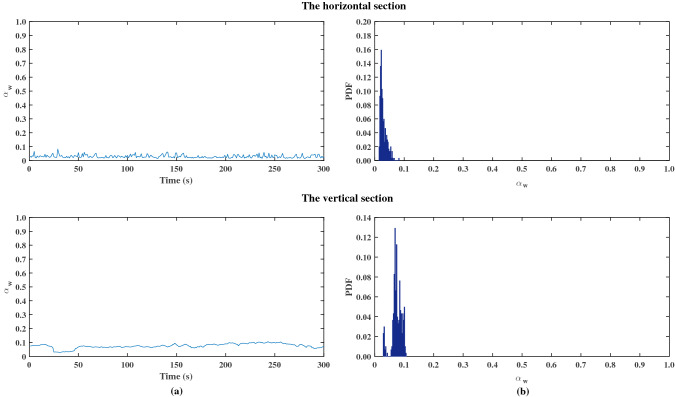
Typical evolution of the cross-sectional average water holdup $\alpha_{{\text{w}}}$ and PDF for oil–water pipe flow: dispersion of water in oil ($u_{{s{\text{w}}}} = 0.02$ m/s, $u_{{s{\text{o}}}} = 0.20$ m/s).

Figures 4 and 5 shows two examples of time series of the cross-sectional average water holdup $\alpha_{{\text{w}}}$ and the corresponding PDF as a function of the water holdup for different experimental conditions. The PDF shows a single narrow peak in both the horizontal and vertical sections, which indicates a small variance in water holdup. This is consistent with the characteristics of the dispersed flow and stratified flow^2,13^. Figure 4 shows the data for $u_{{s{\text{w}}}} = 0.16$ m/s and $u_{{s{\text{o}}}} = 0.07$ m/s. O/W pattern was observed in the horizontal and vertical sections. The O/W pattern was characterized by one narrow peak in the PDF at $\alpha_{{\text{w}}} = 0.72$ in this case. Figure 5 shows the result for $u_{{s{\text{w}}}} = 0.02$ m/s and $u_{{s{\text{o}}}} = 0.20$ m/s. W/O pattern was observed in the horizontal and vertical sections. The PDF showed a single narrow at a low input water holdup ($\alpha_{{\text{w}}} < 0.10$).

**Figure 6:**
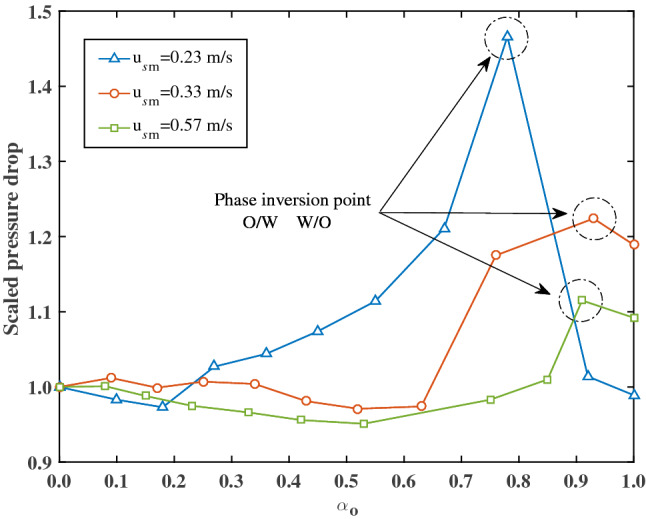
Scaled pressure drop as function of the oil holdup $\alpha_{{\text{o}}}$ for three different total superficial velocities. The phase inversion occurs at an oil holdup between 0.75 and 0.95.

The phase inversion point between O/W and W/O is usually defined as the critical holdup of the dispersed phase above which the dispersed phase becomes the continuous phase. Around the point of phase inversion, the effective mixture viscosity and the pressure drop fluctuate violently^6,15^. Figure 6 shows the scaled pressure drop (ratio of the measured pressure drop and the pressure drop of pure water) as a function of the oil holdup $\alpha_{{\text{o}}}$ for three different total superficial velocities. The pressure drop increases slightly to a maximum value with increasing the oil holdup and then reduces immediately after passes through the maximum. In this study, phase inversion occurs at an oil holdup between 0.75 and 0.95. It can also be seen from Fig. 6 that the pressure drop decreases and the critical oil holdup at phase inversion increases with increasing total superficial velocities.

**Figure 7:**
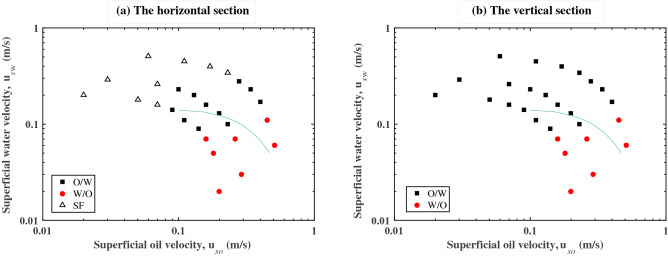
Flow pattern maps of oil–water two-phase pipe flow in (**a**) horizontal section and (**b**) vertical section. $\mu_{{\text{o}}} /\mu_{{\text{w}}} = 32$, $\rho_{{\text{o}}} /\rho_{{\text{w}}} = 0.84$.

The flow pattern maps in terms of superficial velocities of oil and water studied in this paper are demonstrated in Fig. 7. Stratified flow (SF) is observed at high water velocities and low oil velocities in the horizontal section. For decreasing water velocities and increasing oil velocities, the transition to O/W takes place. In the vertical section, only O/W and W/O flow patterns are observed. A comparison between Fig. 7a,b indicates there is a transition from horizontal stratified flow to vertical dispersed flow at the same superficial velocities. The region of O/W flow is larger than that of W/O flow, and the transition boundary from O/W and W/O is almost identical in both the horizontal and vertical sections. The flow patterns in this experiment are obtained with $\mu_{{\text{o}}} /\mu_{{\text{w}}} = 32$ and $\rho_{{\text{o}}} /\rho_{{\text{w}}} = 0.84$.

### Flow pattern transition mechanism

The boundary of the dispersed flow depends on the balance of the turbulent dispersive forces that tend to deform the drop and the surface tension that tend to resist fission. Brauner^16^ proposed a unified approach for predicting the transitions to dispersed flow patterns in gas–liquid and liquid–liquid systems. The transition criteria are based on the part of the work of Hinze^17^. In an oil–water system, the transition to O/W takes place when the water turbulence is strong enough to decompose the oil into droplets smaller than the critical size $d_{{{\text{crit}}}}$:

10 $$ d_{\max } \le d_{{{\text{crit}}}} $$

where *d*_max_ is the maximum diameter that the droplet can reach under the combined action of shear force and surface tension. In dense dispersions, the maximum diameter is given by:

11 $$ \left( {\frac{{d_{\max } }}{D}} \right) = 7.61C_{{\text{H}}}^{0.6} \left( {\frac{{\rho_{{\text{w}}} u_{{s{\text{m}}}}^{2} D}}{\sigma }} \right)^{ - 0.6} \left( {\frac{{\rho_{{\text{w}}} u_{{s{\text{m}}}} D}}{{\mu_{{\text{w}}} }}} \right)^{0.08} \left( {\frac{{u_{{s{\text{o}}}} }}{{u_{{s{\text{w}}}} }}} \right)^{0.6} \left( {1 + \frac{{\rho_{{\text{o}}} u_{{s{\text{o}}}} }}{{\rho_{{\text{w}}} u_{{s{\text{w}}}} }}} \right)^{ - 0.4} $$

where $C_{{\text{H}}}$ is a constant to be determined experimentally, $d_{{{\text{crit}}}}$ refers to the maximum diameter of the droplet without deformation, which can be expressed as:

12 $$ \frac{{d_{{{\text{crit}}}} }}{D} = \frac{0.224}{{\left( {\Delta \rho gD^{2} /8\sigma } \right)^{0.5} }} $$

The scope of application of the above transition criterion is *ρ*_w_u_*s*m_*D*/*µ*_w_ ≥ 2100 and $1.82\left( {{{\rho_{{\text{w}}} u_{{s{\text{m}}}} D} \mathord{\left/ {\vphantom {{\rho_{{\text{w}}} u_{{s{\text{m}}}} D} {\mu_{{\text{w}}} }}} \right. \kern-\nulldelimiterspace} {\mu_{{\text{w}}} }}} \right)^{ - 0.7} < {{d_{{{\text{crit}}}} } \mathord{\left/ {\vphantom {{d_{{{\text{crit}}}} } D}} \right. \kern-\nulldelimiterspace} D} < 0.1$.

The solid line in Fig. 7 represents the boundary that corresponds to the results of the criterion (10). In our experiments, it can better represent the trend from O/W to W/O when $C_{{\text{H}}} = 0.012$. The expression of the critical curve is:

13 $$ \left( {u_{{s{\text{o}}}} + u_{{s{\text{w}}}} } \right)^{1.12} \left( {\frac{{u_{{s{\text{w}}}} }}{{u_{{s{\text{o}}}} }}} \right)^{0.6} \left( {1 + \frac{{\rho_{{\text{o}}} u_{{s{\text{o}}}} }}{{\rho_{{\text{w}}} u_{{s{\text{w}}}} }}} \right)^{0.4} = 0.845\frac{{(\Delta \rho g)^{0.5} \sigma^{0.1} D^{0.48} }}{{\rho_{{\text{w}}}^{0.52} \mu_{{\text{w}}}^{0.08} }} $$

As can be seen in Fig. 7, Eq. (13) can provide somewhat reasonable predictions for phase inversion, although more experimental data are needed to improve the accuracy of the model prediction.

### Phase holdup

**Figure 8:**
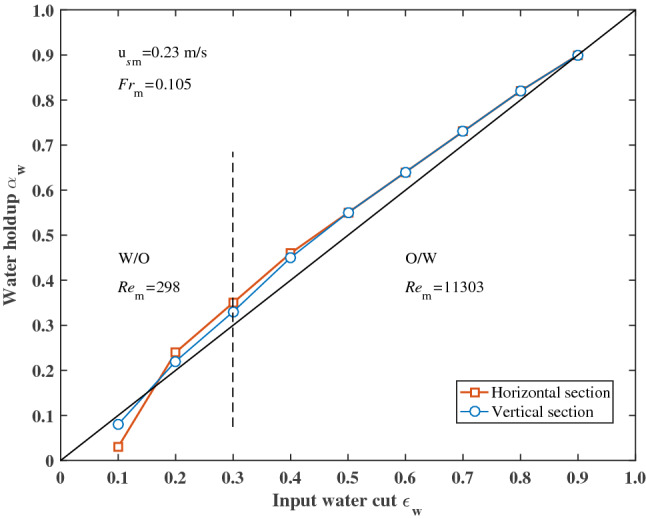
Average water holdup over input water cut at $u_{{s{\text{m}}}} = 0.23$ m/s.

**Figure 9:**
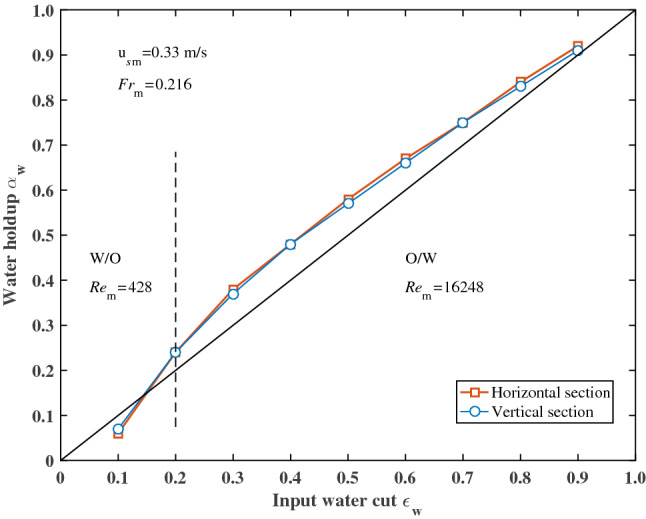
Average water holdup over input water cut at $u_{{s{\text{m}}}} = 0.33$ m/s.

**Figure 10:**
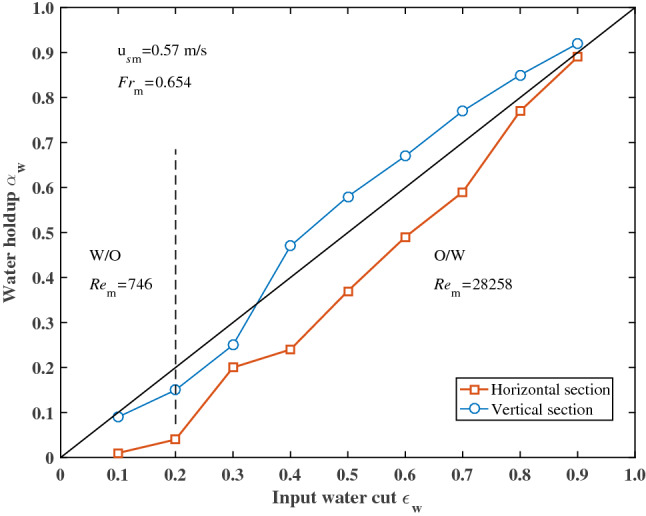
Average water holdup over input water cut at $u_{{s{\text{m}}}} = 0.57$ m/s.

The input water cut has an essential influence on the water holdup in the test section. Figures 8, 9 and 10 shows the average water holdup of the time series in the horizontal and vertical sections at different total superficial velocities (corresponding to different $Re_{{\text{m}}}$ and $Fr_{{\text{m}}}$). The dotted lines in Figs. 8, 9 and 10 represent the boundary between W/O and O/W flow patterns. In the range of W/O, oil forms the continuous phase $Re_{{\text{m}}} = \rho_{{\text{o}}} u_{{s{\text{m}}}} D/\mu_{{\text{o}}}$, whereas, in the range of O/W, water forms the continuous phase $Re_{{\text{m}}} = \rho_{{\text{w}}} u_{{s{\text{m}}}} D/\mu_{{\text{w}}}$. For $Fr_{{\text{m}}} = 0.105$ and 0.216, there are no significant differences in water holdup between horizontal and vertical sections (Figs. 8 and 9). At lower input water cut corresponding to experimental data, the water holdup is slightly lower than the input water cut, i.e., there is a significant velocity difference between the oil and water phases. The range with a smaller water holdup corresponds to the W/O flow pattern range. In the range of the O/W flow pattern, the water holdup is larger than the input water cut. The transition point appears at an input water cut of about 0.2. For $Fr_{{\text{m}}} = 0.654$, the transition point of water holdup from less than input water cut to higher than the input water cut appears at an input water cut of about 0.35 in the vertical section. However, the water holdup is considerably lower than the input water cut in the horizontal section due to water flows faster at this condition (Fig. 10). It also can be seen that for dispersed O/W flow with high input water cut ($\varepsilon_{{\text{w}}} = 0.9$) the dispersed oil droplets travel at approximately the same velocity as the water. The velocity difference between oil and water is close to zero. The result is also similar to that in gas–liquid flow^2^.

The concentration variation of the dispersed phase in the pipe can be predicted by the drift-flux model proposed by Zuber and Findlay^18^. Hasan and Kablr^10^ successfully applied the drift-flux model to the oil–water two-phase flow to predict the concentration of the dispersed phase. For the O/W flow pattern, the drift-flux model calculates the oil holdup:

14 $$ \frac{{u_{{s{\text{o}}}} }}{{\alpha_{{\text{o}}} }} = Cu_{{s{\text{m}}}} + \frac{{J_{d} }}{{\alpha_{o} }} $$

where $J_{d}$ is the drift flux that can be calculated by the following semi-theoretical relationship:

15 $$ J_{d} = u_{\infty } \alpha_{{\text{o}}} \left( {1 - \alpha_{{\text{o}}} } \right)^{n} $$

where *C* and $n$ are parameters determined by experiments, $u_{\infty }$ is the terminal rise velocity of an oil droplet in the static water column, which is calculated by the Harmathy correlation^19^.

16 $$ u_{\infty } = 1.53\left[ {{{\sigma \left( {\rho_{{\text{w}}} - \rho_{{\text{o}}} } \right)g} \mathord{\left/ {\vphantom {{\sigma \left( {\rho_{{\text{w}}} - \rho_{{\text{o}}} } \right)g} {\rho_{{\text{w}}}^{2} }}} \right. \kern-\nulldelimiterspace} {\rho_{{\text{w}}}^{2} }}} \right]^{0.25} $$

Substituting Eq. (15) into Eq. (14) yields the following correlation:

17 $$ {{u_{{s{\text{o}}}} } \mathord{\left/ {\vphantom {{u_{{s{\text{o}}}} } {\alpha_{{\text{o}}} }}} \right. \kern-\nulldelimiterspace} {\alpha_{{\text{o}}} }} = Cu_{{s{\text{m}}}} + u_{\infty } \left( {1 - \alpha_{{\text{o}}} } \right)^{n} $$

Similarly, for the W/O flow pattern, the drift-flux model is transformed into the following form:

18 $$ {{u_{{s{\text{w}}}} } \mathord{\left/ {\vphantom {{u_{{s{\text{w}}}} } {\alpha_{{\text{w}}} }}} \right. \kern-\nulldelimiterspace} {\alpha_{{\text{w}}} }} = Cu_{{s{\text{m}}}} + u_{\infty l} \left( {1 - \alpha_{{\text{w}}} } \right)^{n} $$

where $u_{\infty l}$ is the terminal settlement velocity of a water droplet in static oil column which is calculated by:

19 $$ u_{\infty l} = 1.53\left[ {{{\sigma \left( {\rho_{{\text{w}}} - \rho_{{\text{o}}} } \right)g} \mathord{\left/ {\vphantom {{\sigma \left( {\rho_{{\text{w}}} - \rho_{{\text{o}}} } \right)g} {\rho_{{\text{o}}}^{2} }}} \right. \kern-\nulldelimiterspace} {\rho_{{\text{o}}}^{2} }}} \right]^{0.25} $$

The values of *C* and *n* can be obtained by curve fitting analysis of experimental data. The fitting results for the horizontal and vertical sections are presented in Table 3. The standard deviation (SD) of the predicted value can be expressed as^20^:

20 $$ {\text{SD}} = \sqrt {\frac{1}{n - 1}\sum\limits_{k = 1}^{n} {\left( {\frac{{\left( {\alpha_{D} } \right)_{{{\text{pred}}}} - \left( {\alpha_{D} } \right)_{\exp } }}{{\left( {\alpha_{D} } \right)_{\exp } }}} \right)^{2} } } $$

**Table 3:** The values of *C* and $n$ for experimental data predicted by using the drift-flux model.

Parameter	O/W flow pattern		W/O flow pattern	
	Horizontal section	Vertical section	Horizontal section	Vertical section
*C*	0.65	32.4	–	10.3
*n*	0.17	1.17	–	1.80
Standard deviation (SD)	4.22%	4.40%	–	6.49%

**Figure 11:**
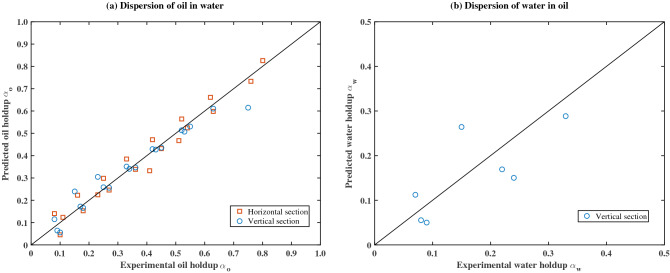
Comparison between the predicted dispersed phase holdup with the experimental data for (**a**) dispersion of oil in water flow pattern and (**b**) dispersion of water in oil flow pattern.

Figure 11 depicts a comparison of the predicted dispersed phase holdup with the experimental data. The square and circular symbols represent the data of the horizontal section and the vertical section, respectively. Better fitting results are obtained for the O/W flow pattern, and the standard deviation of fitting data is within 4.5%. In the range of the W/O flow pattern, a good agreement is obtained for the experimental data of the vertical section at a standard deviation of 6.49%. However, the data in the horizontal section cannot be predicted by the drift-flux model. The reason may be due to the measured holdup data for the horizontal section in the low input water cut are considered to be less reliable. Overall, the existing model provides good estimates of the oil holdup in the dispersion of oil in water flow.

## Conclusion

The oil–water two-phase flow experiments have been conducted in the horizontal and vertical sections of the pipe simultaneously. The difference of oil–water two-phase distribution in horizontal and vertical sections is investigated and discussed. The newly designed water holdup instrument equipped with a conductance probe is used to measure the cross-sectional average water holdup.

The dispersed flow (W/O and O/W) and stratified flow have been identified over the range of oil and water superficial velocities. The pressure drop reaches the maximum value at the phase transition point of O/W and W/O in this study. The phase transition occurs at the oil holdup between 0.75 and 0.95. Based on the model of Brauner^16^, a unified correlation for predicting the transition between O/W and W/O is suggested. The model can provide a reasonable prediction for phase inversion. The drift-flux model proposed by Zuber and Findlay^18^ is modified for predicting the concentration of the dispersed phase in the horizontal and vertical sections. The model can well predict the concentration distribution of the O/W flow patterns. For the W/O flow pattern, the model is suitable for predicting the concentration change in the vertical section but fails to predict the concentration change in the horizontal section.
